# Association of a* BACE1* Gene Polymorphism with Parkinson's Disease in a Norwegian Population

**DOI:** 10.1155/2015/973298

**Published:** 2015-12-14

**Authors:** Johannes Lange, Kristin Aaser Lunde, Camilla Sletten, Simon Geir Møller, Ole-Bjørn Tysnes, Guido Alves, Jan Petter Larsen, Jodi Maple-Grødem

**Affiliations:** ^1^The Norwegian Centre for Movement Disorders, Stavanger University Hospital, 4011 Stavanger, Norway; ^2^Centre for Organelle Research, University of Stavanger, 4036 Stavanger, Norway; ^3^Department of Biological Sciences, St. John's University, New York, NY 11439, USA; ^4^Department of Neurology, Haukeland University Hospital, 5021 Bergen, Norway; ^5^Institute for Clinical Medicine, University of Bergen, 5021 Bergen, Norway; ^6^Department of Neurology, Stavanger University Hospital, 4011 Stavanger, Norway

## Abstract

*Background*. Parkinson's disease (PD) and Alzheimer's disease (AD) share pathological features, including amyloid-beta pathology. Amyloid-beta peptide is generated by sequential proteolysis of amyloid precursor protein (APP), and genetic variations in the processing pathway genes have been found to increase the risk of AD; however, the contribution in PD is unknown.* Methods*. The aim of this study was to investigate whether candidate polymorphisms in five genes (*ADAM10*,* BACE1*,* BACE2*,* PSEN2*, and* CLU*) involved in the APP processing pathway affect PD risk in a population-based cohort of patients with incident PD and control subjects from the Norwegian ParkWest study.* Results*. We found an association of rs638405 in* BACE1* with increased risk of PD, thus providing a novel link, at the genetic level, between amyloid-beta pathology and PD.

## 1. Introduction

Parkinson's disease (PD) and Alzheimer's disease (AD) are the two most common age-related neurodegenerative diseases. PD is primarily a movement disorder, the characteristic motor features of which mainly result from loss of dopaminergic neurons in the substantia nigra pars compacta [1]. However, patients with PD may experience a range of nonmotor symptoms, including some features commonly associated with AD, such as mild cognitive impairment that frequently progresses to dementia [2]. In addition to Lewy body pathology, typical AD related morphological changes, including amyloid-beta (A*β*) pathology, have been proposed to be involved in the pathogenesis of PD dementia. This is reinforced by neuroimaging studies using PET, and with the strong inverse association between A*β* load at autopsy and time to dementia in PD [3, 4].

A*β* peptides are formed by the cleavage of amyloid precursor protein (APP), which can be processed by at least three proteinases, termed *α*-, *β*-, and *γ*-secretases, via either amyloidogenic or nonamyloidogenic APP processing pathways [5]. According to the amyloid cascade hypothesis, an imbalance between A*β* production and clearance plays a critical role in AD pathogenesis. Genetic variations in both* APP* and the genes encoding enzymes of the APP processing pathway are linked to modified risk of AD [6–8], elevated APP production [6], and changes in CSF A*β* levels [9–11]. In light of the occurrence of A*β* pathology in PD, we sought to determine if genetic variations within five genes involved in APP processing and clearance (*ADAM10*,* BACE1*,* BACE2*,* PSEN2*, and* CLU1*) are risk factors for PD in an unselected, population-based cohort of patients with incident PD and age-matched controls.

## 2. Methods

### 2.1. Patients and Controls

All patients (*n* = 184, 60.9% male, mean age at onset 68.1 years ± 9.2 SD) and normal controls (*n* = 178, 51.4% male, mean age at inclusion 66.7 years ± 9.6 SD) participate in the ongoing Norwegian ParkWest study [12]. ParkWest is a large population-based multicenter study of newly diagnosed drug-naïve PD patients designed to determine the incidence, neurobiology, and prognosis of PD. Multiple recruitment strategies were employed to identify all patients with incident PD in four counties in Southwestern Norway during a 22-month period between the 1st of November 2004 and the 31st of August 2006 [12]. Patients underwent comprehensive and standardized clinical examinations before drug treatment was initiated, including rating of disease severity by Unified Parkinson's Disease Rating Scale (UPDRS, baseline score 23.6 ± 11.2 SD) and Hoehn and Yahr staging (baseline score 1.9 ± 0.6 SD). Diagnosis of dementia was made according to the established criteria [13], and by two study neurologists, as previously described [14]. PD patients are currently under continued follow-up, and only those whose diagnosis remained unchanged until their latest clinical visit, on average 4.6 (1.1 SD) years after first presentation, were considered eligible for this study to maximize diagnostic accuracy [12]. During the same time period, we also recruited normal control subjects in the same geographical area. Controls being first- or second-degree relatives of patients were disregarded for single nucleotide polymorphism (SNP) genotype analysis (*n* = 10). All participants signed written-informed consent. The Regional Committee for Research Ethics of the University of Bergen, Norway, approved the study.

### 2.2. Genetic Analyses

To investigate the role of genetic variation in the APP processing genes in PD risk, we first selected loci based on their biologically established role in the APP processing pathway [5]. SNPs were further prioritized if located in a known regulatory or coding region, and/or associated with APP processing or with AD in previous studies (BACE [8, 15]; ADAM10 [9]; PSEN [10]; and CLU [16]). Eight SNPs in 5 genes were selected:* ADAM10* (rs514049, rs2305421),* BACE1* (rs638405, rs11601511),* BACE2* (rs12149, rs2252576),* PSEN2* (rs1295652), and* CLU* (rs11136000). We also included* ApoE* SNPs rs429358 and rs7412, which have been previously linked to PD, AD, and amyloid plaque burden [17–19].

Large-scale allelic discrimination analysis was performed using predesigned TaqMan SNP genotyping assays (Applied Biosystems). The amplification reactions were carried out in an ABI PRISM 7300 Real Time PCR System. Postamplification allelic specific fluorescence was measured using Sequence Detection System software. The call rate for all SNPs was >99%. The concordance rate for replicate TaqMan assays was 100%.

### 2.3. Statistical Analysis

Statistical analyses were computed with the IBM SPSS Statistics 22 software (2013) and the web based tool SNPStats [20]. Statistical significance of differences in genotype and allele frequencies between the patient and control group was assessed with Fisher's exact test (SPSS exact test module). Statistical significance of genotype associations with the risk of either PD or diagnosis of dementia within 5 years of follow-up, odds ratios, and 95% confidence intervals were estimated by logistic regression with age and sex included as covariates (SPSS). Statistical significance of differences of continuous and categorical variables between patient and control group was estimated using *t*-test (age variables), ANCOVA with age and sex included as covariates (continuous variables), or Fisher's exact test (categorical variables). Deviation from Hardy-Weinberg equilibrium was tested using an exact test within the SNPStats tool (with 1 degree of freedom). A value of *P* < 0.05 was considered statistically significant for single comparisons and *P* < 0.006 for multiple comparisons (*n* = 8).

## 3. Results

Blood samples from 184 patients and 178 controls were subject to analysis. The genotype frequencies in patients and controls were in Hardy-Weinberg equilibrium (Table 1). Comparison of genotype frequency between PD and controls showed that the* BACE1* SNP rs638405 was significantly associated with PD (Table 1). Risk of PD association was estimated using logistic regression adjusting for covariates age and gender, showing that the “C” allele conferred increased risk of PD (odds ratio [OR]: 2.29; 95% confidence interval [CI]: 1.24–4.21; *P* = 0.008; see Supplementary Table 1 in Supplementary Material available online at http://dx.doi.org/10.1155/2015/973298). We did not identify an association with rs514049, rs2305421, rs11601511, rs12149, rs2252576, rs1295652, or rs11136000 and risk of PD or clinical measures of PD severity at diagnosis (Table 1 and data not shown).

The* BACE1* rs638405 major allele has been associated with increased risk for late-onset AD (LOAD), and several studies have observed that this effect is enhanced in ApoE-*ε*4 carriers [7, 8, 21, 22]. We tested for a possible link between rs638405 and ApoE-*ε*4 in our cohort and found that ApoE-*ε*4 did not add to the effect of rs638405 on PD risk (data not shown).

Genetic factors that increase A*β* aggregation cause AD and variants in* BACE1* have also been associated with dementia in Down's syndrome [23]. Therefore, we analyzed the possibility that rs638405 is associated with an increased risk of dementia in patients with PD. Of the 184 patients with PD included in this study, 35 were diagnosed with dementia within five years of PD diagnosis. Risk of dementia in patients with PD was estimated using logistic regression adjusting for age and gender. We found no significant association between the “C” allele of rs638405 and the early diagnosis of dementia among the patients with PD ([OR]: 1.64; 95% CI: 0.43–6.29; *P* = 0.471).

## 4. Discussion

This exploratory study shows that rs638405, a* BACE1* SNP previously associated with AD in both Caucasian [7, 8, 21] and Asian populations [22, 24, 25], is also associated with an increased risk of PD in the Norwegian ParkWest study (Table 1). The link between* BACE1* and PD is of clinical importance because of the essential role of BACE1 in the generation of toxic A*β* peptides [26], the observed A*β* pathology in PD [3, 4], and the recent association between detectable changes in A*β* levels and clinical features of PD [14, 27–29].

CSF A*β*42 is emerging as an independent predictor of progression to dementia in patients with PD and is gaining increased attention in the field (reviewed in [30]). In this study, we investigated the usefulness of rs638405 to discriminate PD with dementia from PD without dementia and found no association with the early development of dementia. Research on genetic susceptibility to dementia in PD has been inconclusive; however, several studies have identified SNPs that are associated with dementia in patients with a longer disease course [30], and it will be interesting to reassess the association of* BACE1* SNPs with dementia in more advanced PD.

PD and AD exist along a spectrum of neurodegenerative disorders. The observed overlap in clinical and pathological features has led to the hypothesis that AD and PD may share some common genetic determinants. To investigate this, several studies examined the effect of sporadic PD risk alleles in AD (and vice versa) utilizing GWAS data [31, 32], but they failed to identify loci that increase the risk of both PD and AD. However, studies of targeted candidate SNPs have found associations with both diseases and several loci, including* MAPT* [33, 34],* PON1* [35, 36], and* TREM2* [37], suggesting that some common genetic mechanisms may underlie the pathology of PD and AD.

rs638405 has been repeatedly found to be associated with an increased risk of AD [7, 8, 21, 22, 24, 25] and also with an increased risk of the degenerative neurological disorder sporadic Creutzfeldt-Jakob disease [38]. In light of this, we suggest that although the level of association detected in this study may be due to increased type I errors in multiple comparisons, the* BACE1* rs638405 polymorphism is worthy of further investigation in PD, as it provides both a novel genetic link to the observed overlap in the amyloid pathology of PD and AD and increases the number of neurodegenerative diseases that this variant is associated with. As BACE1 rs638405 is a silent polymorphism, the mechanism of association with disease is unclear. This is further complicated by the findings that the genotype and allele frequencies vary among different ethnic groups, and in different populations either the G allele or the GG genotype [7, 8, 21, 24], or the C allele or CC genotype [22, 25, 38], is associated with increased risk of disease. Some have speculated that this SNP may be in linkage disequilibrium with other functional variant(s), either in the* BACE1* gene itself or in another gene nearby, which have not yet been investigated.

In this study, we targeted our investigation of the genetic overlap between PD and AD by focusing on candidate genes that could contribute to the overlapping A*β* pathology. Limitations of this study include the decision to test only selected genes and SNPs relevant to APP processing and the fact that the cohort size is limited, meaning that some genetic contributions to APP processing and PD risk may have been missed.

A similar approach was recently undertaken in a study of 86 patients with PD and 161 controls, in which a panel of genetic variants in APP processing genes was investigated [39]. The authors did not find an association between* BACE1* polymorphisms and PD compared to controls. However, prior multiple comparison correction results showed an association between APP processing genes and PD compared to controls. Furthermore, the authors showed a significant association between two SNPs in* APP* (rs466448) and* APH1B* (rs2068143) and CSF A*β*42 levels in patients with PD [39]. Taken together, this previous study and the results presented here suggest that variants in APP processing pathway genes may play a biological role in PD.

## 5. Conclusion

In summary, our results show that* BACE1* rs638405 is associated with an increased risk for developing PD, increasing the spectrum of neurodegenerative diseases that this variant plays a role in. These results, together with recently published works showing that A*β* levels are altered in patients with PD in both Norwegian ParkWest study [14, 27, 28] and other studies [29], suggest an interesting area of research, linking common genetic risk factors with AD and overlapping pathogenic mechanisms leading to altered APP processing.

## Supplementary Material

Supplementary Table 1 shows the association of Parkinson's disease risk with genotype estimated using logistic regression adjusting for covariates age and gender.

## Figures and Tables

**Table 1:** Genotype and allele frequencies.

Gene	SNP ID	Allele m/M	*n* PD	*n* controls	Genotype frequencies (*n*)							HWE *P* ^§^
					Patients			Controls			*χ* ^2^ *P* ^#^	
					m/m	m/M	M/M	m/m	m/M	M/M		
*ADAM10*	rs514049	a/C	183	177	32	99	52	27	93	57	0.70	0.11
	rs2305421	g/A	184	178	3	47	134	3	37	138	0.55	1.00

*BACE1*	rs638405	g/C	184	178	19	97	68	35	73	70	0.02	0.91
	rs11601511	c/G	184	178	4	53	127	5	48	125	0.86	1.00

*BACE2*	rs12149	c/T	184	178	48	90	46	41	86	51	0.68	0.60
	rs2252576	t/C	184	178	15	67	102	15	62	101	0.96	0.17

*PSEN2*	rs1295652	a/G	184	178	7	60	117	13	58	107	0.34	0.44

*CLU*	rs11136000	t/C	184	177	28	88	68	23	82	72	0.72	1.00

MAF: minor allele frequency. HWE: Hardy-Weinberg equilibrium. ^**#**^Two-tailed *P* value from Fisher's exact test. ^§^Exact test against Hardy-Weinberg distribution (SNPStats).
