# Supplementary material for: Association of a BACE1 Gene Polymorphism with Parkinson's Disease in a Norwegian Population
Source: Parkinsons Dis. 2015 Dec 14;2015:973298. doi: 10.1155/2015/973298 (PMC4691638; doi:10.1155/2015/973298)
Supplement: Supplementary file 1 — Supplementary Table 1 shows the association of Parkinson's disease risk with genotype estimated using logistic regression adjusting for covariates age and gender. [file 973298.f1.docx]

***Supplementary Table 1 –*** *Genotype and allele frequencies*

|  |  |  |  |  |  | Allele frequencies | | | | | | | | |  | risk of PD association | | | | | | | |
| --- | --- | --- | --- | --- | --- | --- | --- | --- | --- | --- | --- | --- | --- | --- | --- | --- | --- | --- | --- | --- | --- | --- | --- |
|  |  | Allele | n | n |  | MAF | | | | |  | | | |  |  | | | |  | | | |
| Gene | SNP ID | m/M | PD | controls |  | PD | | controls | | | χ^2^ *P^#^* | | | |  | Association *P* | | | | OR (95% CI) | | | |
|  |  |  |  |  |  |  | |  | | |  | | | |  |  | | | |  | | | |
| *ADAM10* | rs514049 | a/C | 183 | 177 |  | 0.45 | | 0.42 | | | 0.45 | | | |  | A allele: 0.57 | | | | 1.14 (0.73-1.80) | | | |
|  |  |  |  |  |  |  | |  | | |  | | | |  | C allele: 0.78 | | | | 0.92 (0.52-1.63) | | | |
|  |  |  |  |  |  |  | |  | | |  | | | |  |  | | | |  | | | |
| *ADAM10* | rs2305421 | g/A | 184 | 178 |  | 0.14 | | 0.12 | | | 0.38 | | | |  | G allele: 0.42 | | | | 1.22 (0.75-1.98) | | | |
|  |  |  |  |  |  |  | |  | | |  | | | |  | A allele: 0.83 | | | | 1.19 (0.23-6.10) | | | |
|  |  |  |  |  |  |  | |  | | |  | | | |  |  | | | |  | | | |
| *BACE1* | rs638405 | g/C | 184 | 178 |  | 0.37 | | 0.40 | | | 0.36 | | | |  | G allele: 0.68 | | | | 1.09 (0.71-1.68) | | | |
|  |  |  |  |  |  |  | |  | | |  | | | |  | C allele: 0.008 | | | | 2.29 (1.24-4.21) | | | |
|  |  |  |  |  |  |  | |  | | |  | | | |  |  | | | |  | | | |
| *BACE1* | rs11601511 | c/G | 184 | 178 |  | 0.17 | | 0.16 | | | 0.92 | | | |  | C allele: 0.62 | | | | 1.12 (0.71-1.77) | | | |
|  |  |  |  |  |  |  | |  | | |  | | | |  | G allele: 0.69 | | | | 1.32 (0.34-5.06) | | | |
|  |  |  |  |  |  |  | |  | | |  | | | |  |  | | | |  | | | |
| *BACE2* | rs12149 | c/T | 184 | 178 |  | 0.51 | | 0.47 | | | 0.37 | | | |  | C allele: 0.48 | | | | 1.18 (0.74-1.89) | | | |
|  |  |  |  |  |  |  | |  | | |  | | | |  | T allele: 0.44 | | | | 0.83 (0.51-1.34) | | | |
|  |  |  |  |  |  |  | |  | | |  | | | |  |  | | | |  | | | |
| *BACE2* | rs2252576 | t/C | 184 | 178 |  | 0.26 | | 0.26 | | | 0.93 | | | |  | T allele: 0.78 | | | | 1.06 (0.70-1.61) | | | |
|  |  |  |  |  |  |  | |  | | |  | | | |  | C allele: 0.83 | | | | 1.09 (0.51-2.31) | | | |
|  |  |  |  |  |  |  | |  | | |  | | | |  |  | | | |  | | | |
| *PSEN2* | rs1295652 | a/G | 184 | 178 |  | 0.20 | | 0.24 | | | 0.28 | | | |  | A allele: 0.52 | | | | 0.87 (0.57-1.33) | | | |
|  |  |  |  |  |  |  | |  | | |  | | | |  | G allele: 0.13 | | | | 2.10 (0.81-5.45) | | | |
|  |  |  |  |  |  |  | |  | | |  | | | |  |  | | | |  | | | |
| *CLU* | rs11136000 | t/C | 184 | 177 |  | 0.39 | | 0.36 | | | 0.44 | | | |  | T allele: 0.47 | | | | 1.17 (0.76-1.79) | | | |
|  |  |  |  |  |  |  | |  | | |  | | | |  | C allele: 0.57 | | | | 0.84 (0.46-1.53) | | | |
|  |  |  |  |  |  | |  | |  |  | |  |  |  | | |  |  |  | |  |  |  |

**MAF**: Minor allele frequency. Risk of PD associations were estimated using logistic regression adjusting for age and gender and a dominant model: allele carrier versus non-carrier. **#** Two-tailed *P* value from Fisher’s exact test.
